# Bis[μ-*N*-(*tert*-butyl­dimethyl­sil­yl)quinolin-8-aminato-1:2κ^2^ *N* ^1^,*N* ^8^:*N* ^8^](*N*,*N*,*N*′,*N*′-tetra­methyl­ethane-1,2-diamine-1κ^2^ *N*,*N*′)lithiumsodium

**DOI:** 10.1107/S160053681204576X

**Published:** 2012-11-14

**Authors:** Juan Chen, Li Yuan

**Affiliations:** a Department of Chemistry, Taiyuan Teachers College, Taiyuan 030031, People’s Republic of China; b Technical Center of Shanxi Entry–Exit Inspection and Quarantine Bureau, No. 8 Yifen Strteet, Taiyuan 030024, People’s Republic of China

## Abstract

In the heterometallic title bulky amido complex, [LiNa(C_15_H_21_N_2_Si)_2_(C_6_H_16_N_2_)], both alkali metal ions are four-coordinated with distorted tetra­hedral geometries. The Li^+^ ion is *N*,*N*′-chelated by the *N*-silylated amido ligand, with Li—N = 2.015 (5) and 2.074 (5) Å. The two amido ligands are arranged *cis* to each other. The mol­ecule exhibits a twofold rotational symmetry operation along the Li–Na axis. The Na^+^ ion is coordinated by two N atoms from the tetra­methyl­ethylenediamine ligand [Na—N = 2.553 (4) Å] and shares two amido N atoms from the *N*-silylated amido ligands with the Li^+^ ion. Although the crystal structure contains voids with an approximate volume of 50 Å^3^ there is no inclusion of solvent mol­ecules.

## Related literature

For related metal complexes with *N-*silylated quinolyl amido ligands, see: Engelhardt *et al.* (1988 ▶, 1990 ▶, 1991 ▶). For silyl-bridged amino­quinoline derivatives, see: Jones *et al.* (2000 ▶). For mixed alkali metal systems as superbase reagents, see: Forbes *et al.* (2003 ▶); Mulvey (2006 ▶); Wei *et al.* (2008 ▶).
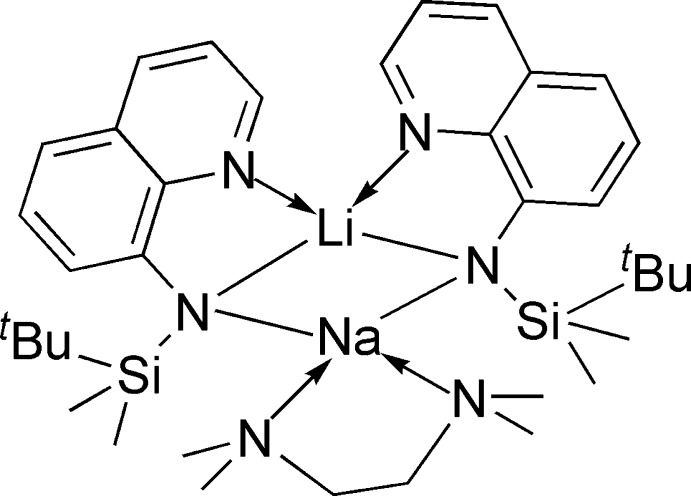


## Experimental

### 

#### Crystal data

[LiNa(C_15_H_21_N_2_Si)_2_(C_6_H_16_N_2_)]
*M* *_r_* = 660.99
Monoclinic, 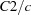

*a* = 12.653 (2) Å
*b* = 18.542 (3) Å
*c* = 18.296 (3) Å
β = 108.794 (3)°
*V* = 4063.6 (11) Å^3^
*Z* = 4
Mo *K*α radiation
μ = 0.13 mm^−1^
*T* = 295 K
0.30 × 0.25 × 0.20 mm

#### Data collection

Bruker SMART CCD diffractometer
Absorption correction: multi-scan (*SADABS*; Sheldrick, 1996 ▶) *T*_min_ = 0.962, *T*_max_ = 0.975
11843 measured reflections
4001 independent reflections
2128 reflections with *I* > 2σ(*I*)
*R*_int_ = 0.071

#### Refinement

*R*[*F* ^2^ > 2σ(*F* ^2^)] = 0.062
*wR*(*F* ^2^) = 0.195
*S* = 0.97
4001 reflections
209 parameters
1 restraint
H-atom parameters constrained
Δρ_max_ = 0.32 e Å^−3^
Δρ_min_ = −0.26 e Å^−3^

### 

Data collection: *SMART* (Bruker, 2000 ▶); cell refinement: *SAINT* (Bruker, 2000 ▶); data reduction: *SAINT*; program(s) used to solve structure: *SHELXS97* (Sheldrick, 2008 ▶); program(s) used to refine structure: *SHELXL97* (Sheldrick, 2008 ▶); molecular graphics: *SHELXTL* (Sheldrick, 2008 ▶); software used to prepare material for publication: *SHELXL97*.

## Supplementary Material

Crystal structure: contains datablock(s) I, global. DOI: 10.1107/S160053681204576X/rk2385sup1.cif

Structure factors: contains datablock(s) I. DOI: 10.1107/S160053681204576X/rk2385Isup2.hkl

Additional supplementary materials:  crystallographic information; 3D view; checkCIF report

## Figures and Tables

**Fig. 1.**
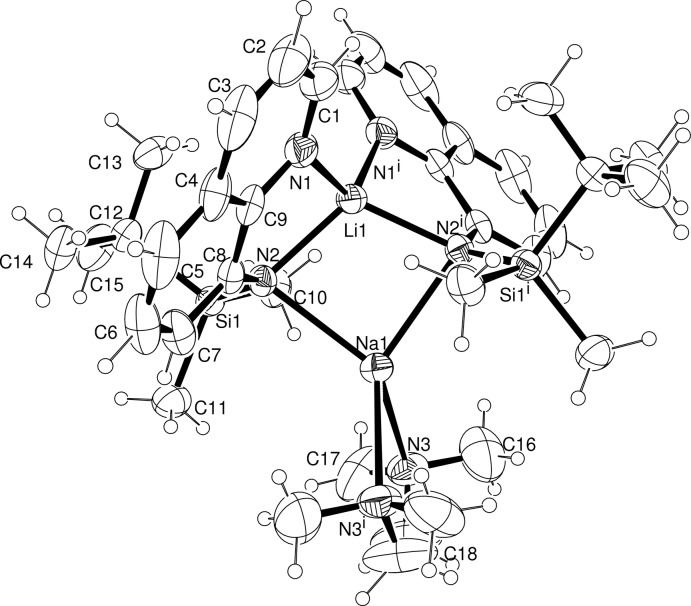
The molecular structure, showing the atom–numbering scheme. Displacement ellipsoids are drawn at the 30% probability level. H atoms are presented as a small spheres of arbitrary radius. Symmetry code: (i) -x+2, y, -z+1/2.

## supplementary crystallographic information

### Comment

8–aminoquinoline is a good chelate amido ligand precursor. It could yield a tetranuclear lithium complex after reacting with *n*–Li*Bu* in *Et*_2_O. The product has a centrosymmetric dimeric structure with a Li_4_N_4_ step–ladder arrangement (Jones *et al.*, 2000). The silylated aminoquinoline, HN(8–C_9_H_6_N)(Si*Me*_3_), is a bulky ligand and it has proven to be suitable to coordinate to Li, Mg, Zn and Al ions (Engelhardt *et al.*, 1988; 1990; 1991). The corresponding lithium complexes exist in either monomeric or dimeric form. The silyl–bridged aminoquinoline turn into a tetradentate ligand and its lithium derivative is tetranuclear (Jones *et al.*, 2000).

The research involving mixed organo–alkali metal amides is vivid as they could serve as superbase reagents and have interesting structures (Forbes *et al.*, 2003; Mulvey, 2006; Wei *et al.*, 2008). Based on the above work, we employed the bulky more demanding aminoquinoline analogue [HN(8–C_9_H_6_N)(Si*Bu^t^Me*_2_)] to prepare a Li/Na hetero alkali metal amide. Its crystal structure is described here.

The title compound was prepared by metallation of the amine with half an equivalent of *n*–butyl lithium and half an equivalent of butyl sodium. Neutral donor *TMEDA* was added into the mixture and the red crystalline product was grown from hexane.

In the molecule of title compound, the lithium ion is fixed by two equivalents of the chelating quinolyl amido ligand, the corresponding bite angle N_amido_–Li–N_quinolyl_ being 84.77 (12)°. The observed Li–N_amido_ bond distance of 2.074 (5)Å is marginally different from reported values in literature and slightly longer than the Li–N_quinolyl_ bond (2.015 (5)Å). It results a distorted tetrahedral configuration around the lithium ion. The sodium atom is connected by the amido nitrogen atoms and it is bound to the neutral donor *TMEDA* simultaneously, which also leads to a distorted tetrahedral geometry. The molecule exhibits a *C*_2_ rotational symmetrical operation along the axis crossing Li and Na atoms. It makes the [Li–N_amido_–Na–N_amido_] cyclic ring to be planar. The two metal atoms are separated by the distance of 2.951 (7)Å.

### Experimental

A solution of 8–*tert*–butyldimethylsilylaminoquinoline (0.71 g, 2.73 mmol) in *Et*_2_O (*ca* 20 ml) was added into the mixture of *n*–Li*Bu* (1.6 *M*, 0.86 ml, 1.37 mmol) and *n*–Na*Bu* (0.11 g, 1.37 mmol) in *Et*_2_O (*ca* 20 ml) at 195 K. Then *TMEDA* (0.16 g, 1.37 mmol) was added and the resulting mixture was kept stirring overnight. The red solution was concentrated and the residue was recrystallized with hexane to give the title compound as red crystals (yield 0.35 g, 39%). ^1^H NMR (300 MHz, C_6_D_6_): δ = 8.588 - 6.735 (m, 12H; quinoline ring), 1.906, 1.840 (d, 16H; *TMEDA*), 1.326, 1.028 (d, 18H; *t*–butyl), 0.497, 0.277 (d, 12H; methyls on silyl); ^13^C NMR (75 MHz, C_6_D_6_): δ = 147.452, 137.652, 136.399, 121.771, 115.578, 110.415 (CH of quinoline ring), 57.652, 46.140 *TMEDA*, 29.237, 26.889 (methyl of *t*–butyl), 18.664, 15.921 (*ipso*–C of *t*–butyl), -0.242, -4.122 (methyls on silyl). Anal. Calc. for C_36_H_58_LiN_6_NaSi_2_: C, 65.42; H, 8.84; N, 12.71%. Found: C, 65.10; H, 8.81; N, 12.69%.

### Refinement

The methyl H atoms were constrained to an ideal geometry, with C—H distances of 0.96Å and *U*_iso_(H) = 1.5*U*_eq_(C), but each group was allowed to rotate freely about its C—C, C—N and C—Si bonds. The methylene H atoms were constrained with C—H distances of 0.97Å and *U*_iso_(H) = 1.2*U*_eq_(C). The quinolyl H atoms were placed in geometrically idealized positions and constrained to ride on their parent atoms, with C—H distances in the range 0.93Å and *U*_iso_(H) = 1.2*U*_eq_(C).

The crystal structure contains four voids (*V* = 48Å^3^) with coordinates: 0.000, 0.373, 0.250; 0.000, 0.627, 0.750; 0.500, 0.873, 0.250; 0.500, 0.127, 0.750. Inclusion of solvent molecules into the voids was not supported by diffraction experiment.

### Crystal data

**Table:**

[LiNa(C_15_H_21_N_2_Si)_2_(C_6_H_16_N_2_)]	*F*(000) = 1432
*M**_r_* = 660.99	*D*_x_ = 1.080 Mg m^−^^3^
Monoclinic, *C*2/*c*	Mo *K*α radiation, λ = 0.71073 Å
Hall symbol: -C 2yc	Cell parameters from 3078 reflections
*a* = 12.653 (2) Å	θ = 2.2–25.5°
*b* = 18.542 (3) Å	µ = 0.13 mm^−^^1^
*c* = 18.296 (3) Å	*T* = 295 K
β = 108.794 (3)°	Block, red
*V* = 4063.6 (11) Å^3^	0.30 × 0.25 × 0.20 mm
*Z* = 4	

### Data collection

**Table:**

Bruker SMART CCD diffractometer	4001 independent reflections
Radiation source: fine-focus sealed tube	2128 reflections with *I* > 2σ(*I*)
Graphite monochromator	*R*_int_ = 0.071
φ and ω scans	θ_max_ = 26.0°, θ_min_ = 2.0°
Absorption correction: multi-scan (*SADABS*; Sheldrick, 1996)	*h* = −8→15
*T*_min_ = 0.962, *T*_max_ = 0.975	*k* = −22→22
11843 measured reflections	*l* = −22→22

### Refinement

**Table:**

Refinement on *F*^2^	Primary atom site location: structure-invariant direct methods
Least-squares matrix: full	Secondary atom site location: difference Fourier map
*R*[*F*^2^ > 2σ(*F*^2^)] = 0.062	Hydrogen site location: inferred from neighbouring sites
*wR*(*F*^2^) = 0.195	H-atom parameters constrained
*S* = 0.97	*w* = 1/[σ^2^(*F*_o_^2^) + (0.1027*P*)^2^] where *P* = (*F*_o_^2^ + 2*F*_c_^2^)/3
4001 reflections	(Δ/σ)_max_ = 0.011
209 parameters	Δρ_max_ = 0.32 e Å^−^^3^
1 restraint	Δρ_min_ = −0.26 e Å^−^^3^

### Special details

**Table:**

Geometry. All esds (except the esd in the dihedral angle between two l.s. planes) are estimated using the full covariance matrix. The cell esds are taken into account individually in the estimation of esds in distances, angles and torsion angles; correlations between esds in cell parameters are only used when they are defined by crystal symmetry. An approximate (isotropic) treatment of cell esds is used for estimating esds involving l.s. planes.
Refinement. Refinement of *F*^2^ against ALL reflections. The weighted *R*–factor *wR* and goodness of fit *S* are based on *F*^2^, conventional *R*–factors *R* are based on *F*, with *F* set to zero for negative *F*^2^. The threshold expression of *F*^2^ > σ(*F*^2^) is used only for calculating *R*–factors(gt) *etc*. and is not relevant to the choice of reflections for refinement. *R*–factors based on *F*^2^ are statistically about twice as large as those based on *F*, and *R*–factors based on ALL data will be even larger.

### Fractional atomic coordinates and isotropic or equivalent isotropic displacement parameters (Å^2^)

**Table:**

	*x*	*y*	*z*	*U*_iso_*/*U*_eq_	
Si1	0.79825 (8)	0.03304 (5)	0.11244 (5)	0.0488 (3)	
Na1	1.0000	−0.04676 (9)	0.2500	0.0575 (5)	
Li1	1.0000	0.1121 (4)	0.2500	0.0521 (19)	
N1	0.9134 (3)	0.16649 (16)	0.30734 (17)	0.0624 (8)	
N2	0.8563 (2)	0.05043 (13)	0.20820 (14)	0.0464 (7)	
N3	1.0507 (4)	−0.15746 (18)	0.1852 (2)	0.0823 (11)	
C1	0.9395 (4)	0.2245 (2)	0.3513 (2)	0.0823 (13)	
H1A	0.9985	0.2530	0.3483	0.099*	
C2	0.8829 (5)	0.2453 (3)	0.4023 (3)	0.110 (2)	
H2A	0.9042	0.2862	0.4329	0.132*	
C3	0.7970 (5)	0.2042 (4)	0.4057 (3)	0.108 (2)	
H3A	0.7591	0.2170	0.4397	0.130*	
C4	0.7628 (4)	0.1428 (3)	0.3595 (2)	0.0729 (12)	
C5	0.6742 (4)	0.1006 (4)	0.3607 (3)	0.1021 (18)	
H5	0.6337	0.1117	0.3936	0.122*	
C6	0.6462 (4)	0.0414 (3)	0.3125 (3)	0.0976 (17)	
H6	0.5867	0.0124	0.3134	0.117*	
C7	0.7050 (3)	0.0245 (2)	0.2627 (2)	0.0748 (12)	
H7	0.6828	−0.0158	0.2311	0.090*	
C8	0.7963 (3)	0.06445 (18)	0.25710 (18)	0.0504 (8)	
C9	0.8254 (3)	0.1260 (2)	0.30898 (18)	0.0545 (9)	
C10	0.9163 (3)	0.0301 (2)	0.0717 (2)	0.0705 (11)	
H10A	0.8932	0.0517	0.0214	0.106*	
H10B	0.9374	−0.0192	0.0679	0.106*	
H10C	0.9789	0.0561	0.1050	0.106*	
C11	0.7207 (4)	−0.05573 (19)	0.0867 (2)	0.0783 (13)	
H11A	0.6527	−0.0483	0.0447	0.117*	
H11B	0.7035	−0.0738	0.1307	0.117*	
H11C	0.7666	−0.0900	0.0715	0.117*	
C12	0.6967 (3)	0.10601 (18)	0.05847 (19)	0.0565 (9)	
C13	0.7530 (4)	0.1797 (2)	0.0801 (3)	0.0890 (14)	
H13A	0.7026	0.2170	0.0534	0.134*	
H13B	0.8195	0.1814	0.0657	0.134*	
H13C	0.7721	0.1869	0.1348	0.134*	
C14	0.5904 (3)	0.1066 (2)	0.0806 (2)	0.0827 (13)	
H14A	0.5416	0.1439	0.0525	0.124*	
H14B	0.6091	0.1154	0.1350	0.124*	
H14C	0.5537	0.0607	0.0683	0.124*	
C15	0.6644 (4)	0.0951 (3)	−0.0291 (2)	0.0963 (16)	
H15A	0.6132	0.1323	−0.0551	0.144*	
H15B	0.6295	0.0489	−0.0426	0.144*	
H15C	0.7301	0.0974	−0.0443	0.144*	
C16	1.1708 (6)	−0.1574 (4)	0.2048 (4)	0.154 (3)	
H16A	1.1936	−0.1983	0.1814	0.231*	
H16B	1.2042	−0.1601	0.2598	0.231*	
H16C	1.1942	−0.1139	0.1861	0.231*	
C17	1.0020 (6)	−0.1651 (3)	0.1025 (3)	0.142 (3)	
H17A	1.0289	−0.2087	0.0862	0.213*	
H17B	1.0226	−0.1246	0.0774	0.213*	
H17C	0.9222	−0.1673	0.0889	0.213*	
C18	1.0183 (8)	−0.2176 (3)	0.2183 (4)	0.168 (3)	
H18A	0.9594	−0.2404	0.1771	0.201*	
H18B	1.0813	−0.2504	0.2310	0.201*	

### Atomic displacement parameters (Å^2^)

**Table:**

	*U*^11^	*U*^22^	*U*^33^	*U*^12^	*U*^13^	*U*^23^
Si1	0.0479 (6)	0.0477 (5)	0.0494 (5)	0.0042 (4)	0.0139 (4)	−0.0013 (4)
Na1	0.0601 (12)	0.0466 (10)	0.0622 (11)	0.000	0.0148 (10)	0.000
Li1	0.046 (5)	0.046 (4)	0.067 (5)	0.000	0.022 (4)	0.000
N1	0.057 (2)	0.0589 (18)	0.0668 (19)	0.0077 (16)	0.0129 (16)	−0.0110 (15)
N2	0.0419 (15)	0.0502 (15)	0.0484 (15)	0.0008 (12)	0.0166 (13)	−0.0002 (12)
N3	0.104 (3)	0.056 (2)	0.085 (3)	0.005 (2)	0.028 (2)	−0.0130 (17)
C1	0.078 (3)	0.071 (3)	0.088 (3)	0.015 (2)	0.011 (3)	−0.025 (2)
C2	0.102 (4)	0.113 (4)	0.090 (4)	0.046 (4)	−0.004 (4)	−0.044 (3)
C3	0.092 (4)	0.171 (6)	0.051 (3)	0.068 (4)	0.008 (3)	−0.022 (3)
C4	0.055 (3)	0.123 (4)	0.039 (2)	0.033 (3)	0.0122 (19)	0.005 (2)
C5	0.069 (3)	0.193 (6)	0.049 (3)	0.034 (4)	0.027 (3)	0.023 (3)
C6	0.052 (3)	0.178 (5)	0.067 (3)	0.001 (3)	0.025 (2)	0.046 (3)
C7	0.057 (2)	0.106 (3)	0.058 (2)	−0.011 (2)	0.015 (2)	0.017 (2)
C8	0.0383 (19)	0.065 (2)	0.0451 (18)	0.0073 (16)	0.0099 (16)	0.0119 (16)
C9	0.044 (2)	0.073 (2)	0.0430 (18)	0.0215 (18)	0.0091 (17)	0.0048 (16)
C10	0.065 (3)	0.086 (3)	0.062 (2)	0.014 (2)	0.024 (2)	−0.0095 (19)
C11	0.092 (3)	0.061 (2)	0.069 (2)	−0.003 (2)	0.008 (2)	−0.0072 (19)
C12	0.057 (2)	0.061 (2)	0.051 (2)	0.0083 (18)	0.0166 (18)	0.0067 (16)
C13	0.093 (3)	0.059 (2)	0.113 (3)	0.014 (2)	0.030 (3)	0.020 (2)
C14	0.061 (3)	0.101 (3)	0.083 (3)	0.023 (2)	0.019 (2)	0.021 (2)
C15	0.105 (4)	0.119 (4)	0.054 (2)	0.037 (3)	0.012 (3)	0.014 (2)
C16	0.114 (5)	0.159 (6)	0.172 (6)	0.003 (5)	0.023 (5)	−0.084 (5)
C17	0.183 (7)	0.135 (5)	0.087 (4)	0.072 (5)	0.015 (4)	−0.026 (3)
C18	0.278 (10)	0.063 (3)	0.199 (8)	0.010 (5)	0.127 (7)	−0.021 (4)

### Geometric parameters (Å, º)

**Table:**

Si1—N2	1.698 (3)	C5—H5	0.9300
Si1—C10	1.873 (4)	C6—C7	1.386 (6)
Si1—C11	1.896 (4)	C6—H6	0.9300
Si1—C12	1.908 (3)	C7—C8	1.403 (5)
Si1—Na1	3.3014 (12)	C7—H7	0.9300
Na1—N2^i^	2.498 (3)	C8—C9	1.454 (5)
Na1—N2	2.498 (3)	C10—H10A	0.9600
Na1—N3	2.553 (4)	C10—H10B	0.9600
Na1—N3^i^	2.553 (4)	C10—H10C	0.9600
Na1—Li1	2.946 (7)	C11—H11A	0.9600
Na1—Si1^i^	3.3014 (12)	C11—H11B	0.9600
Li1—N1^i^	2.015 (5)	C11—H11C	0.9600
Li1—N1	2.015 (5)	C12—C14	1.524 (5)
Li1—N2	2.074 (5)	C12—C15	1.533 (5)
Li1—N2^i^	2.074 (5)	C12—C13	1.533 (5)
Li1—C9^i^	2.765 (4)	C13—H13A	0.9600
Li1—C9	2.765 (4)	C13—H13B	0.9600
Li1—C8	2.768 (4)	C13—H13C	0.9600
Li1—C8^i^	2.768 (4)	C14—H14A	0.9600
N1—C1	1.321 (4)	C14—H14B	0.9600
N1—C9	1.351 (5)	C14—H14C	0.9600
N2—C8	1.373 (4)	C15—H15A	0.9600
N3—C18	1.392 (6)	C15—H15B	0.9600
N3—C16	1.444 (7)	C15—H15C	0.9600
N3—C17	1.446 (5)	C16—H16A	0.9600
C1—C2	1.400 (7)	C16—H16B	0.9600
C1—H1A	0.9300	C16—H16C	0.9600
C2—C3	1.345 (8)	C17—H17A	0.9600
C2—H2A	0.9300	C17—H17B	0.9600
C3—C4	1.401 (7)	C17—H17C	0.9600
C3—H3A	0.9300	C18—C18^i^	1.379 (10)
C4—C5	1.373 (7)	C18—H18A	0.9700
C4—C9	1.432 (5)	C18—H18B	0.9700
C5—C6	1.381 (7)		
			
N2—Si1—C10	106.28 (15)	C3—C2—C1	118.0 (5)
N2—Si1—C11	116.03 (15)	C3—C2—H2A	121.0
C10—Si1—C11	106.90 (18)	C1—C2—H2A	121.0
N2—Si1—C12	113.25 (14)	C2—C3—C4	121.9 (5)
C10—Si1—C12	107.79 (17)	C2—C3—H3A	119.0
C11—Si1—C12	106.18 (17)	C4—C3—H3A	119.0
N2—Si1—Na1	47.72 (9)	C5—C4—C3	123.3 (5)
C10—Si1—Na1	76.71 (12)	C5—C4—C9	120.7 (4)
C11—Si1—Na1	90.67 (12)	C3—C4—C9	116.0 (5)
C12—Si1—Na1	159.86 (11)	C4—C5—C6	119.0 (4)
N2^i^—Na1—N2	87.66 (13)	C4—C5—H5	120.5
N2^i^—Na1—N3	117.08 (11)	C6—C5—H5	120.5
N2—Na1—N3	134.80 (10)	C5—C6—C7	121.3 (5)
N2^i^—Na1—N3^i^	134.80 (10)	C5—C6—H6	119.4
N2—Na1—N3^i^	117.08 (11)	C7—C6—H6	119.4
N3—Na1—N3^i^	72.99 (19)	C6—C7—C8	123.8 (4)
N2^i^—Na1—Li1	43.83 (6)	C6—C7—H7	118.1
N2—Na1—Li1	43.83 (6)	C8—C7—H7	118.1
N3—Na1—Li1	143.51 (9)	N2—C8—C7	126.1 (3)
N3^i^—Na1—Li1	143.51 (9)	N2—C8—C9	119.8 (3)
N2^i^—Na1—Si1^i^	30.20 (6)	C7—C8—C9	114.1 (3)
N2—Na1—Si1^i^	102.57 (8)	N2—C8—Li1	46.37 (17)
N3—Na1—Si1^i^	117.78 (10)	C7—C8—Li1	166.6 (3)
N3^i^—Na1—Si1^i^	104.74 (9)	C9—C8—Li1	74.7 (2)
Li1—Na1—Si1^i^	63.38 (3)	N1—C9—C4	121.6 (4)
N2^i^—Na1—Si1	102.57 (8)	N1—C9—C8	117.3 (3)
N2—Na1—Si1	30.20 (6)	C4—C9—C8	121.0 (4)
N3—Na1—Si1	104.74 (9)	N1—C9—Li1	43.6 (2)
N3^i^—Na1—Si1	117.78 (10)	C4—C9—Li1	161.7 (3)
Li1—Na1—Si1	63.38 (3)	C8—C9—Li1	74.8 (2)
Si1^i^—Na1—Si1	126.75 (6)	Si1—C10—H10A	109.5
N1^i^—Li1—N1	119.9 (4)	Si1—C10—H10B	109.5
N1^i^—Li1—N2	130.03 (11)	H10A—C10—H10B	109.5
N1—Li1—N2	84.77 (12)	Si1—C10—H10C	109.5
N1^i^—Li1—N2^i^	84.77 (12)	H10A—C10—H10C	109.5
N1—Li1—N2^i^	130.03 (11)	H10B—C10—H10C	109.5
N2—Li1—N2^i^	113.1 (4)	Si1—C11—H11A	109.5
N1^i^—Li1—C9^i^	27.53 (11)	Si1—C11—H11B	109.5
N1—Li1—C9^i^	142.5 (3)	H11A—C11—H11B	109.5
N2—Li1—C9^i^	128.53 (19)	Si1—C11—H11C	109.5
N2^i^—Li1—C9^i^	58.66 (11)	H11A—C11—H11C	109.5
N1^i^—Li1—C9	142.5 (3)	H11B—C11—H11C	109.5
N1—Li1—C9	27.53 (11)	C14—C12—C15	108.5 (3)
N2—Li1—C9	58.66 (11)	C14—C12—C13	107.5 (3)
N2^i^—Li1—C9	128.53 (19)	C15—C12—C13	109.4 (3)
C9^i^—Li1—C9	169.3 (3)	C14—C12—Si1	111.8 (2)
N1^i^—Li1—C8	148.81 (15)	C15—C12—Si1	110.8 (3)
N1—Li1—C8	57.60 (12)	C13—C12—Si1	108.6 (3)
N2—Li1—C8	28.62 (10)	C12—C13—H13A	109.5
N2^i^—Li1—C8	121.7 (3)	C12—C13—H13B	109.5
C9^i^—Li1—C8	157.1 (2)	H13A—C13—H13B	109.5
C9—Li1—C8	30.47 (10)	C12—C13—H13C	109.5
N1^i^—Li1—C8^i^	57.60 (12)	H13A—C13—H13C	109.5
N1—Li1—C8^i^	148.81 (15)	H13B—C13—H13C	109.5
N2—Li1—C8^i^	121.7 (3)	C12—C14—H14A	109.5
N2^i^—Li1—C8^i^	28.62 (10)	C12—C14—H14B	109.5
C9^i^—Li1—C8^i^	30.47 (10)	H14A—C14—H14B	109.5
C9—Li1—C8^i^	157.1 (2)	C12—C14—H14C	109.5
C8—Li1—C8^i^	142.7 (3)	H14A—C14—H14C	109.5
N1^i^—Li1—Na1	120.0 (2)	H14B—C14—H14C	109.5
N1—Li1—Na1	120.0 (2)	C12—C15—H15A	109.5
N2—Li1—Na1	56.53 (18)	C12—C15—H15B	109.5
N2^i^—Li1—Na1	56.53 (18)	H15A—C15—H15B	109.5
C9^i^—Li1—Na1	95.34 (17)	C12—C15—H15C	109.5
C9—Li1—Na1	95.34 (17)	H15A—C15—H15C	109.5
C8—Li1—Na1	71.37 (16)	H15B—C15—H15C	109.5
C8^i^—Li1—Na1	71.37 (16)	N3—C16—H16A	109.5
C1—N1—C9	119.0 (4)	N3—C16—H16B	109.5
C1—N1—Li1	130.8 (3)	H16A—C16—H16B	109.5
C9—N1—Li1	108.9 (3)	N3—C16—H16C	109.5
C8—N2—Si1	124.2 (2)	H16A—C16—H16C	109.5
C8—N2—Li1	105.0 (2)	H16B—C16—H16C	109.5
Si1—N2—Li1	121.45 (14)	N3—C17—H17A	109.5
C8—N2—Na1	115.99 (18)	N3—C17—H17B	109.5
Si1—N2—Na1	102.07 (12)	H17A—C17—H17B	109.5
Li1—N2—Na1	79.64 (19)	N3—C17—H17C	109.5
C18—N3—C16	109.0 (6)	H17A—C17—H17C	109.5
C18—N3—C17	106.9 (5)	H17B—C17—H17C	109.5
C16—N3—C17	108.6 (5)	C18^i^—C18—N3	126.3 (3)
C18—N3—Na1	106.8 (3)	C18^i^—C18—H18A	105.8
C16—N3—Na1	106.6 (3)	N3—C18—H18A	105.8
C17—N3—Na1	118.6 (3)	C18^i^—C18—H18B	105.8
N1—C1—C2	123.3 (5)	N3—C18—H18B	105.8
N1—C1—H1A	118.4	H18A—C18—H18B	106.2
C2—C1—H1A	118.4		

Symmetry code: (i) −*x*+2, *y*, −*z*+1/2.
